# Accounting for center-level effects in multicenter randomized controlled trials

**DOI:** 10.1186/s13063-024-08202-w

**Published:** 2024-06-18

**Authors:** Shofiqul Islam, Shrikant I. Bangdiwala

**Affiliations:** 1 Department of Health Research Methods, Evidence and Impact, McMaster University, Hamilton, Canada; 2 Statistics, Population Health Research Institute, Hamilton, Canada

## Abstract

Investigators often conduct randomized controlled trials (RCTs) at multiple centers/sites when determining the effect of a treatment or an intervention. Diversifying recruitment across multiple institutions allows investigators to make recruitment go faster within a shorter timeframe and allows generalizing the study results across diverse populations. Despite having a common study protocol across multiple centers, the eligible participants may be heterogeneous, site policies and practices may vary, and the investigators’ experience, training, and expertise may also vary across sites. These factors may contribute to the heterogeneity in effect estimates across centers. As a result, we usually observe some degree of heterogeneity in effect estimates across centers, despite all centers following the same study protocol. During the analysis of such a trial, investigators typically ignore center effects, but some have suggested considering centers as fixed or random effects in the model. It is not clear how considering the effects of centers, either as fixed or random effects, impacts the test of the primary hypothesis. In this article, we first review the practice of accounting for center effects in the analyses of published RCTs and illustrate the extent of heterogeneity observed in a few preexisting multicenter RCTs. To determine the impact of heterogeneity on the test of a primary hypothesis of an RCT, we considered continuous and binary outcomes and the corresponding appropriate model, namely, a simple linear regression model for a continuous outcome and a logistic regression model for the binary outcome. For each model type, we considered three methods: (a) ignore the center effect, (b) account for centers as fixed effects, or (c) account for centers as random effects. Based on simulation studies of these models, we then examine whether considering the center as a fixed or random effect in the model helps to preserve or reduce the type I and type II error rates during the analysis phase of an RCT. Finally, we outline the threshold at which center-level effects are negligible and thus negligible and provide recommendations on when it may be necessary to account for center effects during the analyses of multicenter randomized controlled trials.

**Supplementary Information:**

The online version contains supplementary material available at 10.1186/s13063-024-08202-w.

## Introduction

Investigators often conduct randomized controlled trials (RCTs) at multiple centers when determining the effect of a treatment or an intervention [1–3]. Multicenter RCTs are conducted when a single center or site may not provide sufficient numbers of potential participants or when investigators at a single site may not be able to manage a large number of participants [4]. Diversifying recruitment across multiple centers also allows investigators to make recruitment go faster within a shorter timeframe. More importantly, this approach allows researchers to generalize the study results across diverse populations and clinical settings. Despite having a common study protocol across multiple sites, eligible participants may be heterogeneous across centers/sites, site policies and practices may vary, and investigators’ experience and staff training and expertise may also vary across sites [5]. All of these factors may contribute to the heterogeneity in effect estimates across centers.

A natural approach to conducting an RCT is to allocate treatment or intervention to participants by randomizing within the center so that the idiosyncrasies of a center are balanced between treatment arms and that there is no problem with internal validity. Since all procedures, training, equipment, and eligibility criteria are standardized across centers, in theory, we do not expect to have substantial heterogeneity in treatment or intervention effects across centers. The treatment or intervention effects in each center are considered unbiased estimates of the true effect. However, the actual effect at a center depends on the center’s compliance with the common protocol as well as on random variation. In practice, we observe some (usually small) heterogeneity in effect estimates across centers; these are called “residual center effects.”

While analyzing a multicenter trial, authors may consider adjusting the heterogeneity in point estimates across different centers. Well-recognized methods of analyzing such trial data include (a) ignoring center effects in analyses [6], (b) accounting for center effects in models using centers as fixed effects [7],John T. [8, 9], or (c) accounting for center effects in models using centers as random effects [3].

The objective of this study was to evaluate under what circumstances one could ignore the differences in estimates of treatment or intervention effects across different centers and, if notable, whether there was a difference in modeling the center as fixed or random effects. We first examine the extent to which multicenter randomized controlled trials (RCTs) published in leading clinical journals address the issue of accounting or ignoring center effects and, if considered, whether they are treated as fixed or random effects in analyses. Second, we illustrated the heterogeneity in center effects in selected multicenter RCTs with different types of outcomes (continuous, binary). Finally, using simulations, we study whether the choice of fixed versus random effects in different models helps to preserve or reduce the type I and type II error rates during the analysis phase of an RCT.

The ultimate objective of this study was to determine if there is a threshold at which center-level effects are negligible or negligible and to provide recommendations for the analysis of future multicenter randomized controlled trials.

## Methods

### Accounting for center effects in published literature

To understand the extent to which multicenter RCTs published in leading clinical journals address the issue of center effects, we searched for RCTs published in the *New England Journal of Medicine* and *The Lancet* from January 2017 to June 2023. The systematic search started with the key phrase “randomized controlled trial” and then was narrowed down to those who indicated “multicenter” or related terms (multicenter or multicentre or multi-centre) in the title or the abstract of the article. We then scanned through the statistical analysis section of those articles to determine the number of articles that considered the center as a fixed or random effect in the model.

### Heterogeneity of center effects in past RCTs

To understand the degree of heterogeneity we may observe in different types of outcomes (continuous, binary) across different centers, we explored a few multicenter randomized controlled trials conducted at the Population Health Research Institute (PHRI). This approach was essential for planning the different scenarios to study in several simulation studies of RCTs.

### Simulation study

To determine the impact of heterogeneity on the test of a statistical hypothesis (difference in the treatment or intervention effects), we considered continuous and binary outcomes and the corresponding appropriate model, namely, a simple linear regression model for a continuous outcome to test the difference in means between two groups and a logistic regression model for the binary outcome to test the difference in proportions of events. For each model type, we considered three methods: (a) ignore the center effect, (b) account for centers as fixed effects, or (c) account for centers as random effects. Model specifications associated with each of these outcomes are provided in Additional file 1: Appendix A. These models allow us to test the null hypothesis that “there is no treatment or intervention effect” (e.g., *β* = 0).

In a standard clinical trial, the study is designed with the expectation that the null hypothesis (e.g., *β* = 0) will be rejected. In other words, the investigators try to determine whether there is some treatment or intervention effect that can be detected with the desired level of significance and power. However, the test results of an RCT can be negative (there is no statistically significant treatment or intervention effect) because of a small treatment/intervention effect, because the study failed to account for correct design-level parameters such as variances, or because the sample size needed to detect a prespecified effect of interest before the trial was underestimated. In a multicenter RCT, the total variance can further be impacted by the variability within or between centers.

At the design phase of a trial, appropriate choices of sample size and variance of the outcome (accounting for between-center and within-center variance components, as reflected in the intraclass correlation coefficient) can have a substantial impact on the ability to detect a clinically meaningful effect. To determine the impact of these choices on the decision to test the null hypothesis, we conducted an extensive simulation study. We compared the type I error rates (the proportion of times the null hypothesis was rejected when the null hypothesis was true) and the type II error rates (the proportion of times the null hypothesis was not rejected when the alternative hypothesis was true) with varying sample sizes, effect estimates, and the intraclass correlation coefficient (ICC). We hypothesized that ignoring the center-level effect during the analysis may lead to a greater probability of type II error (*β*) than what the investigator planned during the design phase of a clinical trial.

Specifying the necessary sample size to detect a prespecified effect and knowing the variance of the outcome variable are critical parameters for making a correct decision in randomized controlled trials. Of these design-level parameters, a correct variance estimate may be the most difficult for an investigator to know at the design stage. In a multicenter RCT, the total variance in the primary outcome could be partitioned into subject-level and center-level variability. We often call these within- and between-center variances the components of the intraclass correlation coefficient. Based on the observed ICCs in various PHRI multicenter trials, we designed and conducted simulation studies to determine the impact of the three different choices of model-fitting options.

A simulation study is expected to be based on specific design-level parameters. The sample size required to test for a clinically meaningful effect of a treatment or intervention in an RCT is usually predetermined based on a variance estimate and the desired probabilities of type I and type II errors [or Power = 1 − Pr (type II error)]. Of these parameters, the correct choice of the total variance at the design phase is key to keeping the desired type I and type II error probabilities under control. However, investigators may fail to recognize the center-level variability in the outcome in a multicenter setup of an RCT. As a result, the total variance can either be over- or underestimated when the trial is being planned. For example, failing to recognize between-center variability may lead to a higher total variance than estimated. The proportion of total variance due to center-level differences is usually calculated based on the ICC.

Incorrect choice of the total variance (between-center and within-center) can impact the calculation of the necessary sample size at the design phase of a trial and can have a substantial impact on the decision of the hypothesis we plan to investigate. We varied effect estimates, sample sizes, and variances (between-center and within-center or within-center) in the simulation. Each sample was generated using a within-center randomization procedure, a standard approach for a multicenter RCT. Using 10,000 simulated samples in each scenario, we calculated rejection rates from the tests associated with each of the three models and plotted them. The parameter specifications for the different scenarios are presented in Additional file 1: Table S1 and are briefly described below.

For a continuous outcome, we considered an RCT to detect 5 units of reduction in systolic blood pressure (SBP in mmHg) in the treatment group compared to the control group. Assuming a total variance of 400 mmHg^2^, a sample of size 600 (treatment 300, control 300) is required to achieve 86% power at a two-sided 5% level of significance [10]. In our hypothetical study, participants were recruited at 12 different centers, with 50 subjects from each center. Assuming a within-center variance of 380 mmHg^2^ and a between-center variance of 20 mmHg^2^, we have a fixed total variance of 400 mmHg^2^ with an ICC = 0.05 [10]. While varying the ICC, we considered two different scenarios that an investigator may encounter after the trial: (1) correct estimation of total variance but higher/lower between-center variability and (2) incorrect estimation of total variance, failing to recognize higher/lower between-center variability. To investigate the effect of the second scenario, we varied the ICC from 0 to 0.3 while keeping the total variance fixed at 400 mmHg^2^, the sample size was fixed at 600, and the effect estimate was 5 mmHg. A figure for a generated sample is provided in the Additional file 1.

For a binary outcome, we considered an RCT to detect an 8% reduction in the incidence of hypertension in the treatment group compared to the control group, assuming a control group incidence of hypertension of 40%. Since the outcome was binary, we fit a logistic regression model, and the variability within the center, in this case, was fixed at π^2^/3 = 3.29. We require a sample of 3600 participants for this study (treatment 1800, control 1800) to achieve 90% power at a two-sided 5% level of significance to detect an odds ratio (OR) or relative risk (RR) of 0.8 (20% reduction in odds of hypertension in the treatment or intervention group compared to the control group) [10]. We also assumed that the investigator would plan to recruit participants from 60 different centers, with 60 participants from each center. Under this assumption, the within-center variance is 3.29, and the between-center variance is 0.1645, which leads to a total variance of 3.46 and an ICC = 0.0475.

We evaluate the results by comparing the three different choices of models, focusing on their type I and type II error rates for testing the null hypothesis with varying ICCs, sample sizes, and treatment or intervention effect estimates.

## Results

### Accounting for center effects in published literature

Our systematic search of the literature in the *New England Journal of Medicine* and *The Lancet* during the last 5 years (January 2017 to July 2023) revealed 274 multicenter randomized controlled trials related to articles (226 in the *New England Journal of Medicine* (NEJM), 48 in *The Lancet*) that used the terms “fixed effect” or “random effect.” Of these, only 16 considered modeling center effects, 5 considered centers as fixed effects in the model, and 11 considered centers as random effects in the model. A summary of this search is provided in Additional file 1: Table S2.

### Heterogeneity of center effects in past RCTs

To utilize sensible parameter estimates in our simulations, we identified three multicenter randomized controlled trials (RCTs) conducted at the Population Health Research Institute (PHRI) for which we had access to individual patient data: the Outcome Reduction with an Initial Glargine Intervention trial (ORIGIN) [11], Heart Outcomes Prevention Evaluation–3 (HOPE3) [12], and Cardiovascular Outcomes for People Using Anticoagulation Strategies (COMPASS) [2]. A summary of these trials in terms of variances and ICCs for a few binary and continuous outcomes is presented in Table 1. We observe that the ICC estimates range between 0.05 and 0.15 for different binary and continuous outcomes.

**Table 1:** Empirical observed between-center variability from three selected RCTs conducted at the Population Health Research Institute, with corresponding observed ICCs for different outcomes

Study name	Number of subjects (centers)	Clinical outcome	Type of outcome	Between center variance	ICC
**ORIGIN**	12,500 (575)	Mortality	Binary	0.243	0.069
		Fasting glucose	Continuous	0.377	0.097
		HbA1c	Continuous	0.0004	0.054
		SBP	Continuous	51.54	0.110
**HOPE-3**	12,705 (223)	Mortality	Binary	0.402	0.109
		CVD: MI/stroke	Binary	0.354	0.097
**COMPASS**	27,389 (602)	BMI	Continuous	3.398	0.150
		Total cholesterol	Continuous	0.145	0.127

### Simulation studies

#### Continuous outcome

**Fig. 1:**
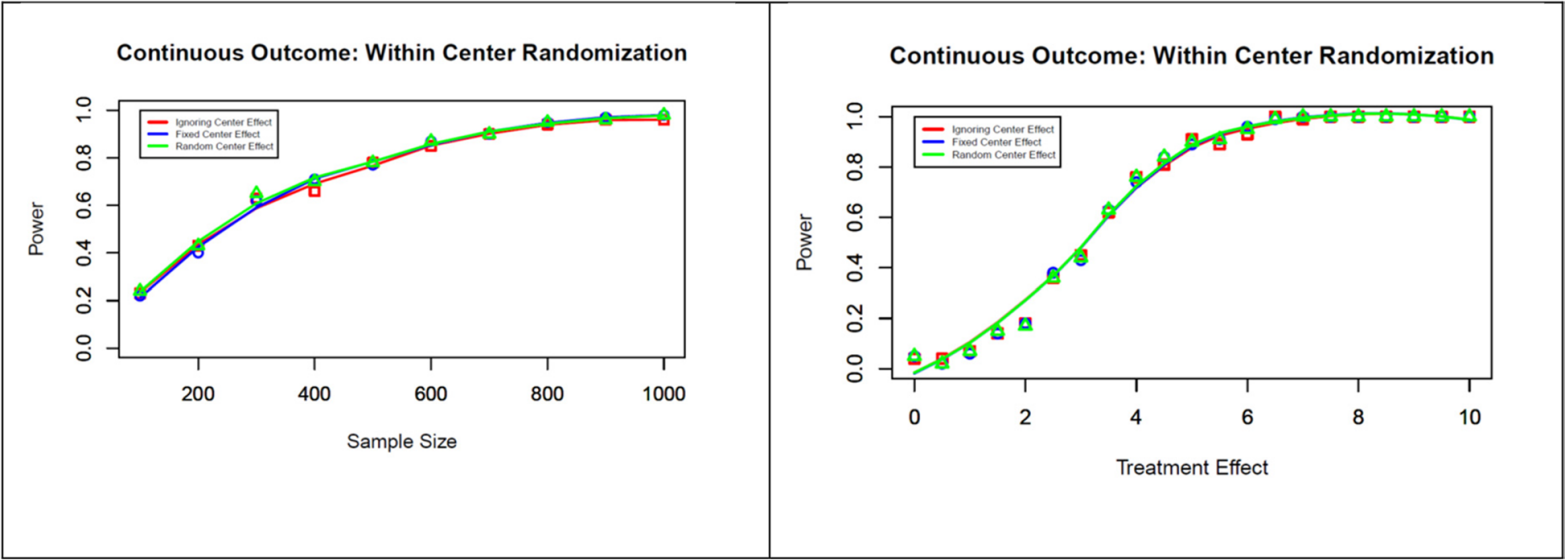
Null hypothesis rejection rates for a continuous outcome using within-center randomization, with varying sample sizes and effect estimates (assumed ICC = 0.05)

**Fig. 2:**
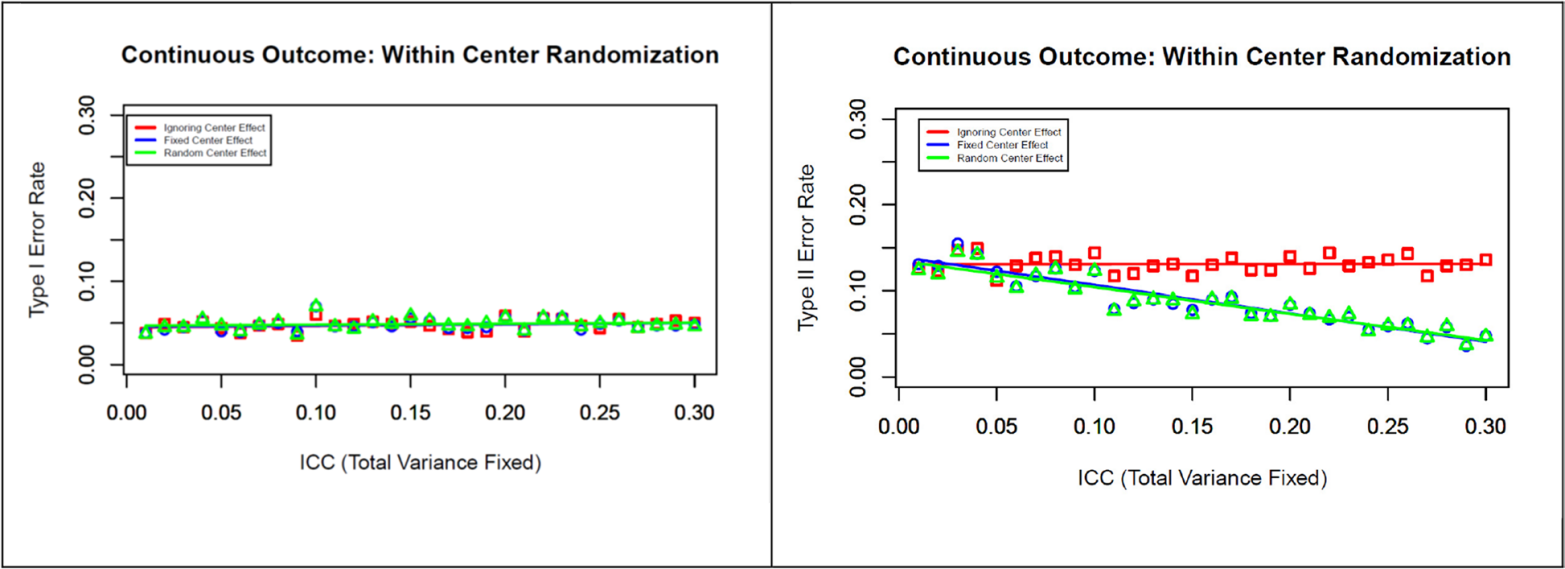
Effects on type I and type II error rates determined via within-center randomization with increasing between-center variability (increasing ICC) but with fixed total variance

**Fig. 3:**
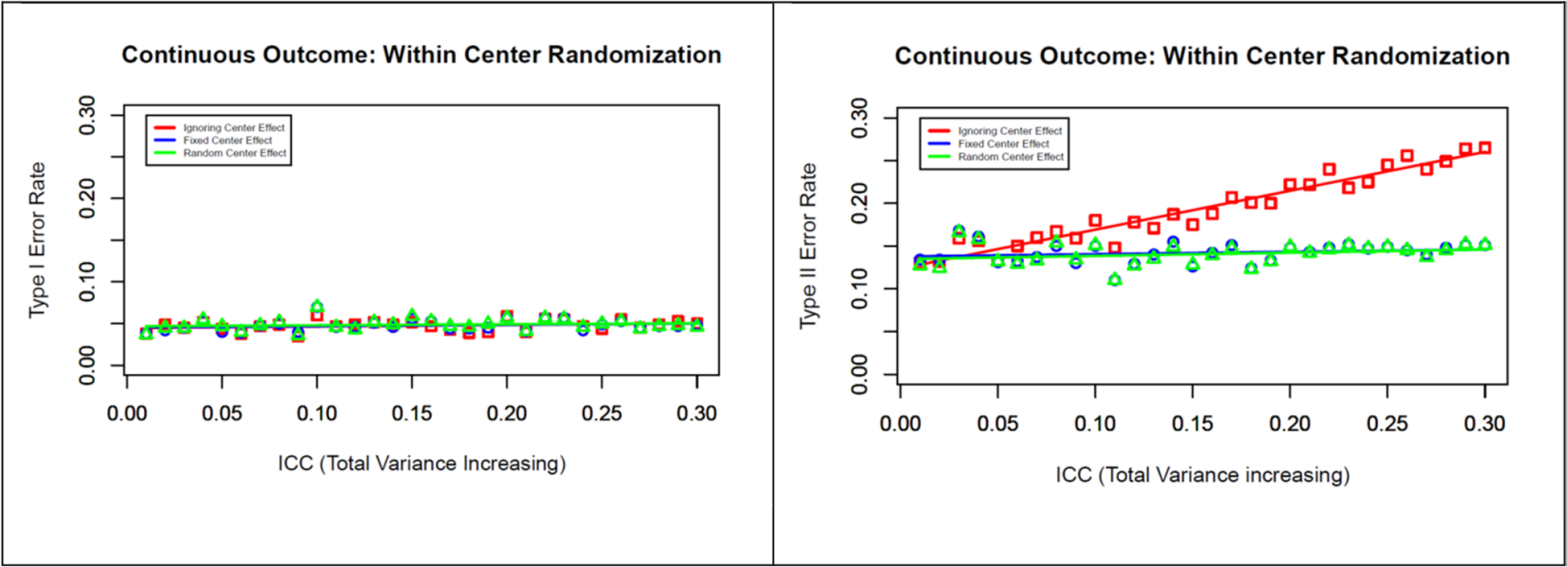
Effects of within-center randomization on type I and type II error rates for a continuous outcome with increasing between-center variability (increasing ICC) but increasing total variance

The simulation study results for a continuous outcome are presented in Figs. 1, 2, and 3. By varying the sample size and keeping the effect estimates at 5 mmHg of SBP and an ICC of 0.05 (left panel of Fig. 1), we were not able to find any difference in type I or type II error rates across the three models. Similarly, we were not able to detect any difference in type I or type II error rates when the effect estimates were varied (0, 1, …, 10 mmHg), keeping the sample size fixed at 600 and the fixed ICC = 0.05 (right panel of Fig. 1).

As noted earlier, we consider two different scenarios while varying the ICC. In the first scenario, we assumed that the investigator determined the sample size before the trial based on the correct total variability but failed to recognize the variability between centers that leads to a greater ICC. The results of this simulation study are presented in Fig. 2. During this simulation, we observe that the random effect model suffers from a singular fit of the covariance matrix in some of the simulated data sets with smaller ICCs (e.g., < 0.05). However, parameter estimates for large ICCs (e.g., > 0.05) provide stable parameter estimates. We are not able to find any difference in type I error rates in three different models (left panel of Fig. 2), but ignoring the center effect in the model seems to preserve the type II error rates around the design level specification (right panel of Fig. 2). However, considering the center either as a fixed or as a random effect in the model helps reduce the type II error rates with increasing ICC.

In the second scenario with varying ICCs, we assumed that the investigator determined the sample size before the trial based on incorrect total variability, failing to recognize the greater between-center variability during the trial. The simulation results for this scenario are presented in Fig. 3. Since the investigator failed to recognize the between-center variability before the trial, the total variance was underestimated, as a greater ICC was observed during the analysis phase of the trial. To investigate the effect of this scenario, we varied the ICC (0, 0.02, 0.03, …, 0.3), thus leading to an increase in total variance (400, 420,…, 520 mmHg^2^), with a fixed sample of size 600 and an effect estimate of 5 mmHg. Like in the previous scenario, we are not able to find any difference in type I error rates among the three different models (left panel of Fig. 3); however, considering the center either as a fixed or as a random effect in the model helps to preserve the type II error rates at the design level (right panel of Fig. 3). It should also be noted that both models performed equally in terms of preserving the type II error rate in this simulation, even with the increasing level of heterogeneity across centers. However, ignoring the center effect in the model leads to increased type II error rates with increasing ICC, thus reducing the overall power of the study.

### Binary outcome

**Fig. 4:**
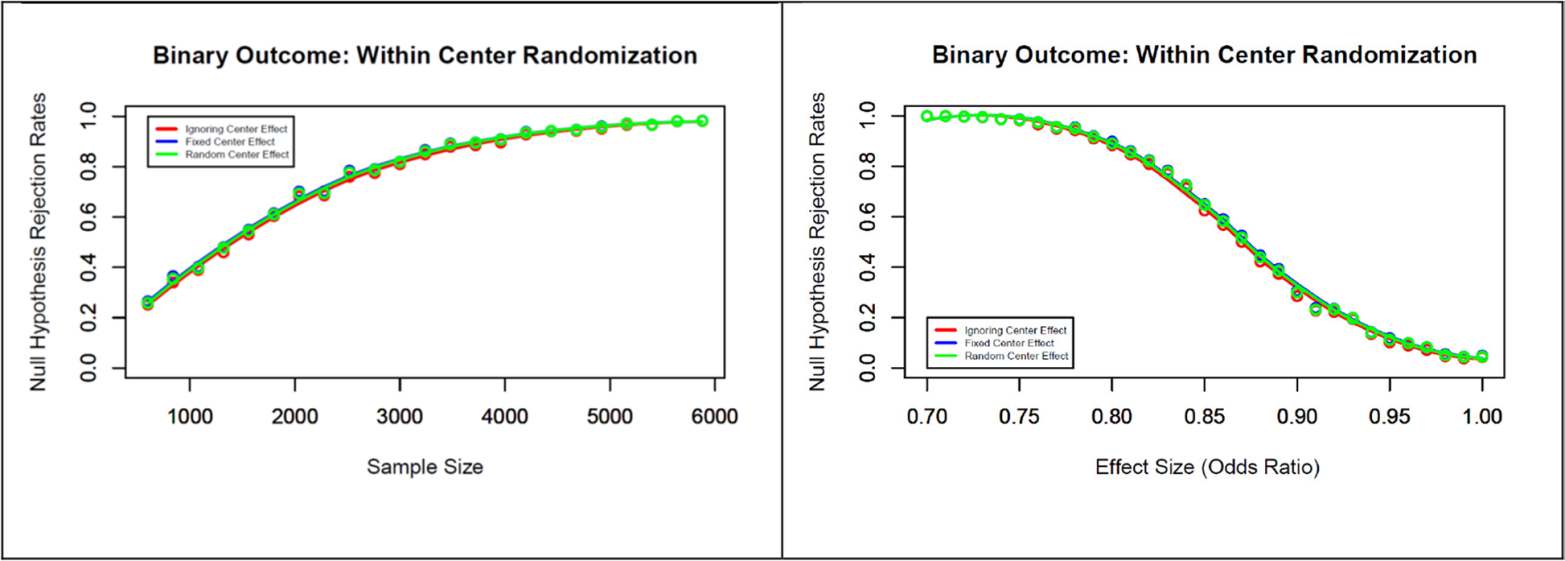
Null hypothesis rejection rates for a binary outcome using within-center randomization with varying sample sizes and effect estimates (assumed to be an ICC of 0.05)

**Fig. 5:**
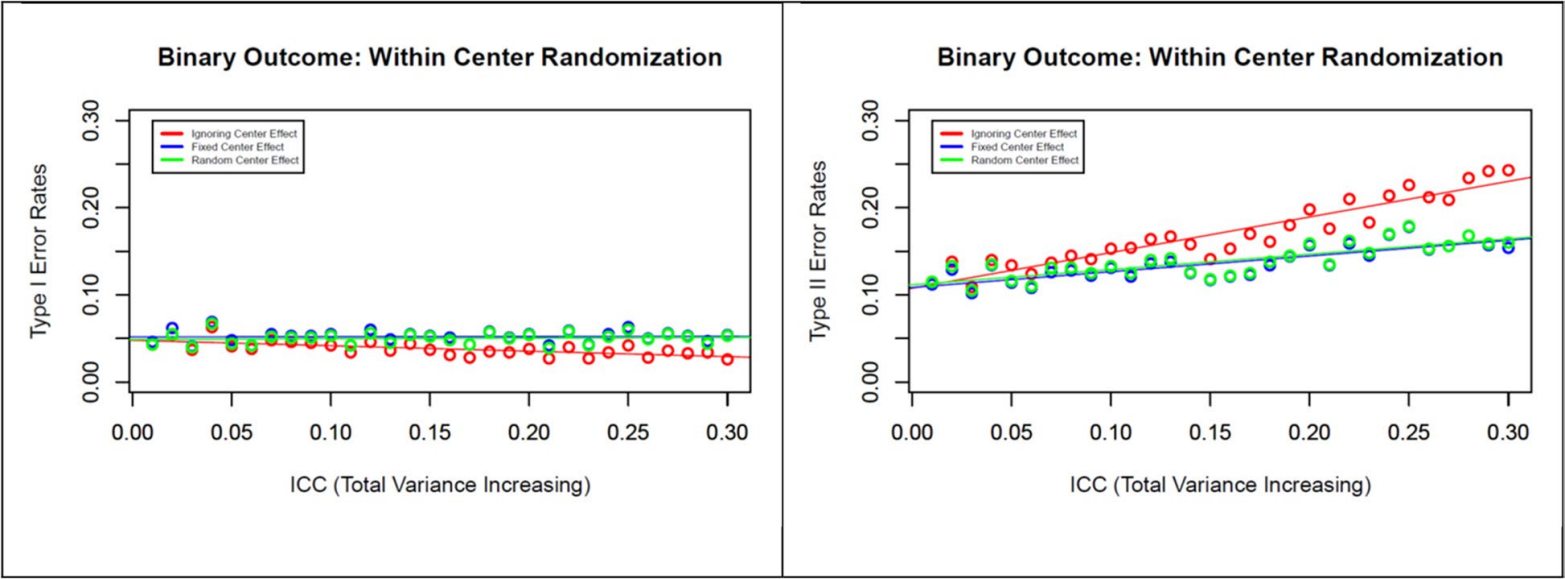
Effects of within-center randomization on type I and type II error rates for a binary outcome with increasing between-center variability (increasing ICC) but with fixed total variance

The simulation study results for a binary outcome are presented in Figs. 4 and 5. By varying the sample size (600, 1200, …, 6000) while keeping the effect estimate of OR or RR = 0.8 and the ICC at 0.05 (left panel of Fig. 4), we were not able to find any difference in type I (no figure included) or type II error rates. Similarly, we were not able to find any difference in type I (no figure included) or type II error rates when the effect estimates were varied (OR or RR = 0.7, 0.72, …, 1.0), keeping the sample size fixed at 3600 and a fixed ICC = 0.05 (right panel of Fig. 4).

While varying the ICC, we assumed that the investigator determined the sample size before the trial based on an incorrect total variance, failing to recognize the higher between-center variability during the trial. To investigate the effect of this scenario, we varied the ICC (0, 0.02, 0.03, …, 0.3), leading to greater total variance (3.46, 3.62, …, 4.28), keeping the sample size fixed at 3600, and the effect estimate at OR or RR = 0.8. The results of this simulation study are presented in Fig. 5. If we ignore the center effect in the model, we observe a minor decreasing tendency in type I error rates with increasing ICC, but this trend remains consistent with the designed level if we include the center as either a fixed or random effect in the model (left panel of Fig. 5). Considering the center either as a fixed or as a random effect in the model helps to preserve the type II error rates at the designed level setup (right panel of Fig. 5). It should also be noted that both models performed equally well in terms of preserving the type II error rate in this simulation, even with the increasing level of heterogeneity across centers. However, ignoring the center effect in the model leads to higher type II error rates with increasing ICC.

When between-center variability is very low (less than 5%), for either continuous or binary outcomes, the random effect model usually suffers from a singular fit of the covariance matrix. As a result, the confidence interval estimate of the treatment effect may not be estimable or reliable. We also do not gain much in terms of preserving or reducing the type I or type II error rate. Thus, we do not recommend using a random effect model when the post hoc ICC estimate is lower than 0.05.

## Discussion

Different choices of models (no center effect, center as fixed, and center as random) seemed to have a notable impact when there was some heterogeneity, especially when there was an underestimate of the total variance in the design phase of the trial. If the total variance estimate is correct, as assumed in the design phase, an increase in between-center variance and the ICC is not harmful. In this case, ignoring the center effect in the model will not negatively impact the study power, but including the center either as a fixed or as a random effect in the model will lead to reduced type II error, which leads to increased power with increasing ICC. On the other hand, if the total variance estimate is not correct (underestimated) at the design phase, an increase in the between-center variability will increase the total variance and hence the ICC. In this situation, ignoring the center effect in the model will lead to reduced power with increasing ICC. However, including the center either as a fixed or as a random effect in the model helps preserve the power specified at the design phase of an RCT.

Based on the simulation study, we are not able to find any major impact on type I or type II error rates with varying sample sizes across three different regression models, and the results are consistent for both binary and continuous outcomes. Similarly, we are not able to find any notable difference in type I and type II error rates with varying effect estimates. The simulation study in this article is restricted to 60 centers. However, the number of centers may be substantially smaller or larger than 60 in an RCT. While the number of centers plays a critical role in a clustered randomized trial (directly proportional to the sample size), it has a negligible effect on a within-center randomization approach, but considering the between-center variability and correctly specifying the total variance is key. It should also be noted that the simulation study in this article is restricted to a continuous and binary outcome only.

**Fig. 6:**
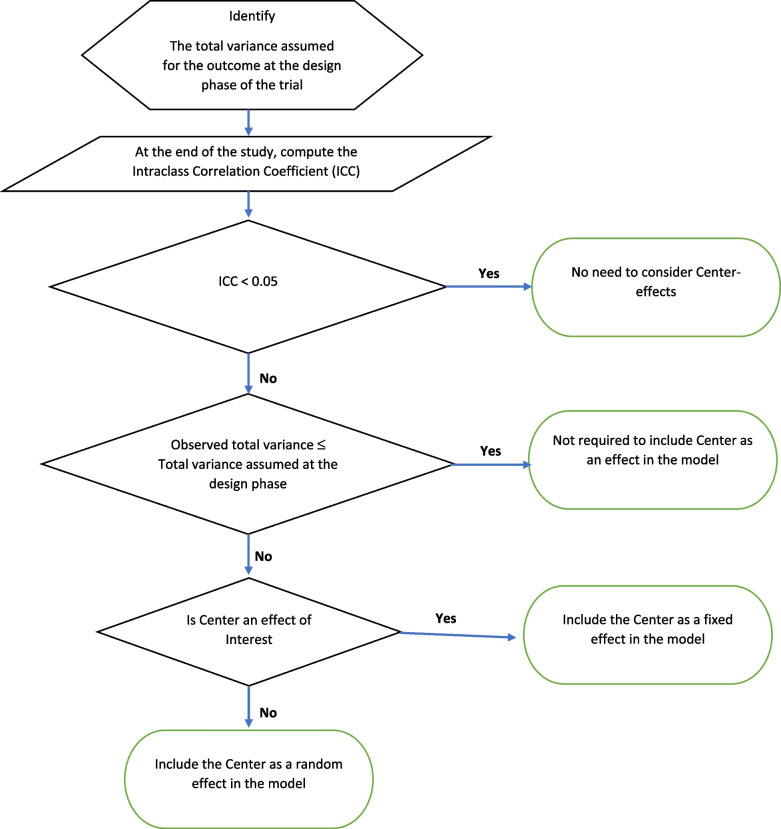
Flow chart: how to determine which model to use

We were not able to find any consensus in the literature on whether we should include the center as an effect in the model during the analysis of multicenter RCT data. Only a few authors have discussed and emphasized the importance of accounting for the central effect in a multicenter randomized controlled trial [9, 13], but our literature review in the *New England Journal of Medicine* (NEJM) and *The Lancet* showed that a very small proportion of trials considered this topic. Finally, our simulation study shows identical performance when considering either the center as a fixed or as a random effect in the model, preserving or reducing the type II error rate for both binary and continuous outcomes. However, choosing between fixed vs random requires additional consideration. In the context of binary response, Agresti et al. discussed in detail under which circumstances one should choose fixed vs random effect models [14]. For example, they have indicated that although centers are not usually selected randomly in a multicenter RCT, many share the view that a random effects approach still better reflects all the actual sources of variability. We also recommend using the center as a random effect in the model unless the investigator is interested in identifying centers with notable differences in treatment efficacy, especially if the number of centers is large. A flow chart determining which model to use during the analysis phase of the trial is presented in Fig. 6.

## Conclusion and recommendations

The analysis of three multicenter RCTs revealed that a moderate degree of heterogeneity likely existed across centers for different outcomes [ICC ranged from 0.05 to 0.15], despite all studies having a common protocol and standard, uniform operating procedures at all centers. The simulation study results showed that, in the presence of a small degree of heterogeneity (e.g., ICC < 0.05), we could safely ignore the center as a fixed or random effect in the model. If the variability between centers is high but the observed total variance is similar to the total variance estimate assumed before the trial, ignoring the center effect will still preserve the desired type I and type II error rates. However, based on our simulation study (Fig. 2), we recommend including the center either as a fixed or random effect in the model, as this approach helps reduce the type II error rate. If the variability between centers is greater (e.g., greater than 5% of the total variability) and leads to a higher total variance estimate than the design level set-up, considering the center as a random effect in the model will help to preserve the correct type I and type II error rates during the analysis phase of the trial.

In summary, we recommend designing an RCT considering the correct variance estimate that is directly related to the power of the test of a statistical hypothesis. At the end of the trial, we recommend calculating the total variance (partitioning within- and between-center variance) as well as the ICC. If the total variance is greater than the design-level setup, failing to recognize between-center variability, consider including the center as a random effect in the model to avoid unusually higher type II error rates for testing the primary hypothesis of interest.

### Supplementary Information

Additional file 1: Appendix A. Table S1. Table S2. Figure S1. Figure S2.

## Data Availability

The article is mainly based on a simulation study. Small secondary data sets used from different trials to produce Table 1 are not available to upload in the public domain.
