# Supplementary material for: Accounting for center-level effects in multicenter randomized controlled trials
Source: Trials. 2024 Jun 18;25:390. doi: 10.1186/s13063-024-08202-w (PMC11184780; doi:10.1186/s13063-024-08202-w)
Supplement: Supplementary file 1 — Additional file 1: Appendix A. Table S1. Table S2. Figure S1. Figure S2. [file 13063_2024_8202_MOESM1_ESM.docx]

**Accounting for center-level effects in multicenter randomized control trials**

**Shofiqul Islam^[[1]](#footnote-1)^ and Shrikant I Bangdiwala**

**Appendix A:**

**Models associated with a continuous or binary outcome are presented below:**

**Continuous outcome:**

Let us suppose *Y_ij_* is a continuous outcome corresponding to the *i^th^* subject and *j^th^* center. $\theta_{j}$ (fixed) or *γ _j_* (random) represents the *j^th^* center-level effects. *T_ij_* is an indicator variable, that represents the treatment or intervention to which the *i^th^* subject from the *j^th^* center has been allocated. Thus, models associated with a continuous outcome can be written as:

**No center-effect:**

$$Y_{ij}=\alpha+\beta T_{ij}+ e_{ij}, i=1,2,3\ldots n_{i}, j=1,2,3,\ldots,J ,$$

where $e_{ij} are i.i.d. \sim N\left( 0,\sigma_{e}^{2} \right)$.

**Fixed center-effect:**

$Y_{ij}=\alpha+ \beta T_{ij}+\theta_{j} C_{j}+ e_{ij}, i=1,2,3\ldots n_{i}, j=1,2,3,\ldots,J$,

where $e_{ij} are i.i.d. \sim N\left( 0,\sigma_{e}^{2} \right)$ and $C_{j}$ is a dummy indicator variable for the *j^th^* center.

**Random center-effect:**

$$Y_{ij}=\alpha+ \beta T_{ij}+\gamma_{j}+ e_{ij}, where i=1,2,3\ldots n_{i}, j=1,2,3,\ldots,J,$$

where $e_{ij}\sim N\left( 0,\sigma_{w}^{2} \right) and \gamma_{j} \sim N\left( 0,\sigma_{b}^{2} \right)$ are independent.

**Binary outcome:**

Assuming $p_{i}$ as the probability of an event given treatment or intervention and using similar notations, models associated with a binary outcome can be written as:

1. **No center-effect:**

$$\log(\frac{p_{i}}{1-p_{i}})=\alpha+ \beta T_{ij}, i=1,2,3\ldots n_{i}, j=1,2,3,\ldots,J$$

2. **Fixed center-effect:**

$$\log(\frac{p_{i}}{1-p_{i}})=\alpha+ \beta T_{ij}+\theta_{j} C_{j}, i=1,2,3\ldots n_{i}, j=1,2,3,\ldots,J$$

where $C_{j}$ is a dummy indicator variable for the *j^th^* center.

1. **Random center-effect:**

$$\log(\frac{p_{i}}{1-p_{i}})=\alpha+ \beta T_{ij}+\gamma_{j}, i=1,2,3\ldots n_{i}, j=1,2,3,\ldots,J$$

where$\gamma_{j} \sim N\left( 0,\sigma_{b}^{2} \right)$.

**Appendix Table 1: Simulation Scenarios**

| **Simulation Parameters** | |
| --- | --- |
| **Continuous Outcome: Blood Pressure (BP)** | **Binary Outcome: Hypertension** |
| **Vary effect estimates**   - Reduction in Blood Pressure (BP): 0, 1, 2, …, 10 mmHg - Number of centers: 12 (50 subjects/center) - ICC = 0.05 - Type I error rate = 0.05; Type II Error Rate = 0.14 | **Vary effect estimates**   - Odds Ratio (Treatment vs Hypertension): 0.7, 0.72, …, 1.0 - Number of centers: 60 (60 subjects/center) - ICC = 0.05 - Type I error rate = 0.05; Type II Error Rate = 0.10 |
| **Vary sample size by a varying number of centers**   - Effect estimate: 5 mmHg - Number of centers: 2, 4, 6, …, 20 (50 subjects/center) - ICC = 0.05 - Type I error rate = 0.05; Type II Error Rate = 0.14 | **Vary sample size by a varying number of centers**   - Effect estimate: Odds Ratio = 0.8 - Number of centers: 10, 20, 30, …, 100 (60 subjects/center) - ICC = 0.05 - Type I error rate = 0.05; Type II Error Rate = 0.10 |
| **Vary the distribution of variances and corresponding ICC**   - Effect estimate: 5 mmHg - Consider the ICC: 0.01, 0.02, …, 0.3   - Fixed total variance but with increasing ICC   - Increasing total variance with increasing ICC - Number of centers: 12 (50 subjects/center) - Type I error rate = 0.05; Type II Error Rate = 0.14 | **Vary the distribution of variances and corresponding ICC**   - Effect estimate: Odds Ratio = 0.8 - Consider the ICC: 0.01, 0.02, …, 0.3   - Increasing total variance with increasing ICC - Number of centers: 60 (60 subjects/center) - Type I error rate = 0.05; Type II Error Rate = 0.10 |

**Appendix Table 2: Summary of current literature search**

| **Search criteria: ‘randomized controlled trial’ in the New England Journal of Medicine and the Lancet during last five years, from January 01, 2017 to June 15, 2023** |
| --- |
| 1. New England Journal of Medicine.jn (128486) 2. Lancet.jn. (468620) 3. Randomized controlled **traial** (1074425) 4. Multicenter/Multi-center/Multicentre/Multi-centere (539134) 5. Year 2017 – today (June 15, 2023) 6. Random effect/Random **center** effect (516120) 7. Fixed effect/ Fixed **center** effect (1250817) 8. 1 and 3 and 4 and 5 and 6 (135) 9. 1 and 3 and 4 and 5 and 7 (91) 10. 2 and 3 and 4 and 5 and 6 (36) 11. 2 and 3 and 4 and 5 and 7 (12) |

**Appendix Figure 1: Probability density curve of systolic blood pressure (SBP) by the control and intervention group from a simulated sample.**


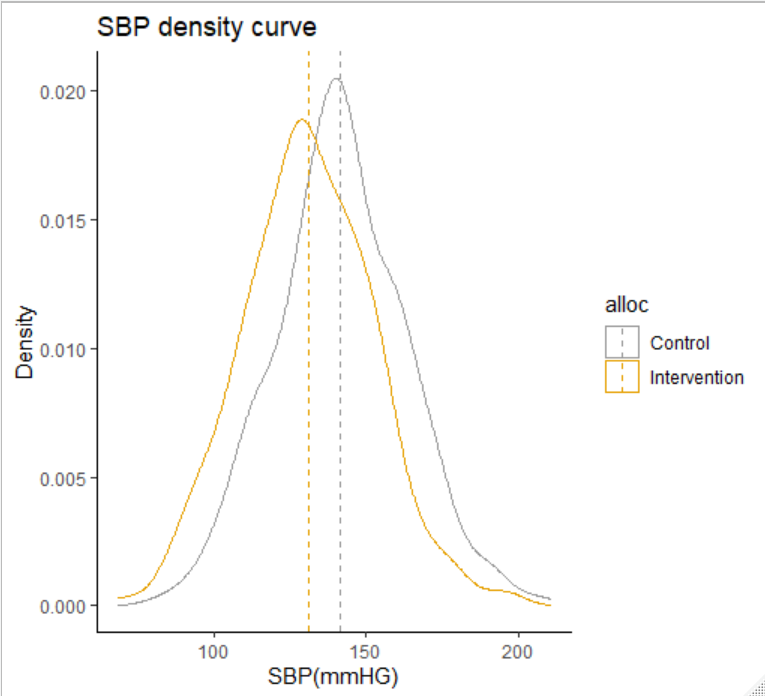


**Appendix Figure 2: Effects on Type I and Type II error rates using within-center randomization with increasing between-center variability (Increasing ICC) but with fixed total variance.**

**[ Same as Figure 2 in the article but X-axis restricted up to 0.10 ICC]**

|  | 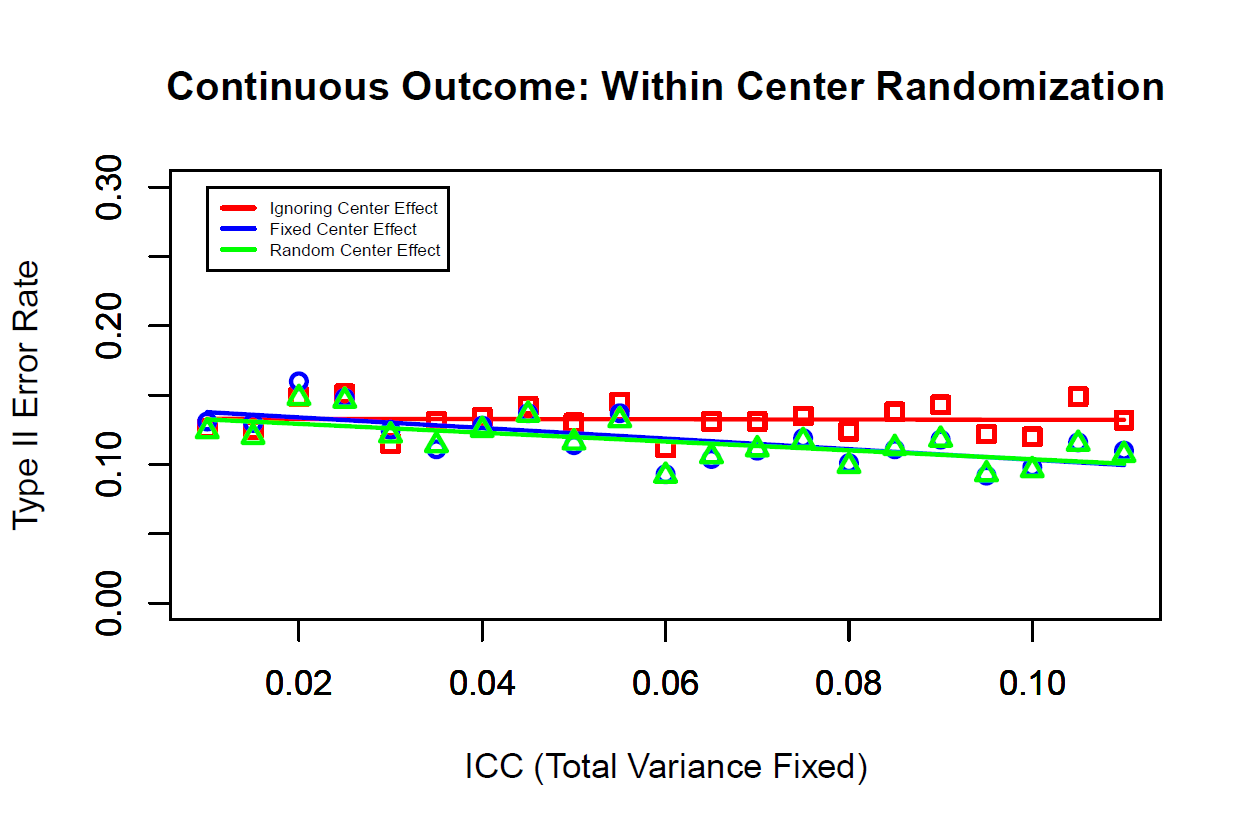 |
| --- | --- |

1. Corresponding author:

   Shofiqul Islam, Department of Health Research Methods, Evidence and Impact, McMaster University [↑](#footnote-ref-1)
